# Dramatic dysbalancing of the Wnt pathway in breast cancers

**DOI:** 10.1038/s41598-018-25672-6

**Published:** 2018-05-09

**Authors:** Alexey Koval, Vladimir L. Katanaev

**Affiliations:** 1 Department of Pharmacology and Toxicology, Faculty of Biology and Medicine, University of Lausanne, Lausanne, 1011 Switzerland; 2 School of Biomedicine, Far Eastern Federal University, Vladivostok, Russian Federation

## Abstract

Wnt signaling is important for breast development and remodeling during pregnancy and lactation. Epigenetic modifications change expression levels of components of the Wnt pathway, underlying oncogenic transformation. However, no clear Wnt component increasing expression universally across breast cancer (BC) or its most Wnt-dependent triple-negative BC (TNBC) subgroup has been identified, delaying development of targeted therapies. Here we perform network correlation analysis of expression of >100 Wnt pathway components in hundreds of healthy and cancerous breast tissues. Varying in expression levels among people, Wnt components remarkably coordinate their production; this coordination is dramatically decreased in BC. Clusters with coordinated gene expression exist within the healthy cohort, highlighting Wnt signaling subtypes. Different BC subgroups are identified, characterized by different remaining Wnt signaling signatures, providing the rational for patient stratification for personalizing the therapeutic applications. Key pairwise interactions within the Wnt pathway (some inherited and some established *de novo*) emerge as targets for future drug discovery against BC.

## Introduction

Aberrant activation of the Wnt-FZD signaling pathway leads to tumorigenesis in many tissues^1,2^ including the breast^3,4^. Nineteen members of the Wnt family of secreted lipoglycoproteins and ten members of the FZD family of seven transmembrane-helix receptors are encoded by the human genome. Signaling in the canonical pathway promoting cell proliferation relies on reorganization of the Axin/APC/GSK3β destruction complex, functioning to promote degradation of the cytoplasmic pool of β-catenin. In the presence of Wnt ligands, FZD and the co-receptor LRP5/6, through the joint action of the scaffolding protein Dishevelled and heterotrimeric G proteins, induce reorganization of the complex resulting in stabilization of the cytoplasmic β-catenin, which can enter the nucleus and activate transcription of the Wnt target genes through interactions with TCF and other co-factors^5,6^. Stabilization of β-catenin can be seen in BC and a variety of other cancers^1,7^. In addition to the canonical pathway, β-catenin-independent Wnt pathways have been described to control cytoskeleton, and through this – cell motility and cancer metastasis^8^.

The generally assumed picture concerning the alteration of the Wnt pathway during breast transformation is that, through epigenetic changes, expression of positive components of the canonical Wnt pathway is increased, while expression of negative components or antagonists of this pathway is decreased^3,4^. Of the positive canonical components, examples of proposed over-expressions include: canonical Wnt ligand proteins^9,10^ (e.g. Wnt1 and Wnt10b), receptors^11,12^ (LRP6, FZD7), and transducers^13,14^ (e.g. Dvl1, Tcf4). Some proposed under-expressions include non-canonical Wnts^15,16^ (e.g. Wnt5a, Wnt5b), secreted antagonist proteins^17–19^ (e.g. sFRP1, sFRP5, WIF1), and negative transducers^20,21^ (e.g. NKD2, APC). These works painted the picture of general overactivation of the canonical Wnt pathway in BC and proposed certain Wnt pathway components as potential targets for targeted drug discovery.

**Figure 1:**
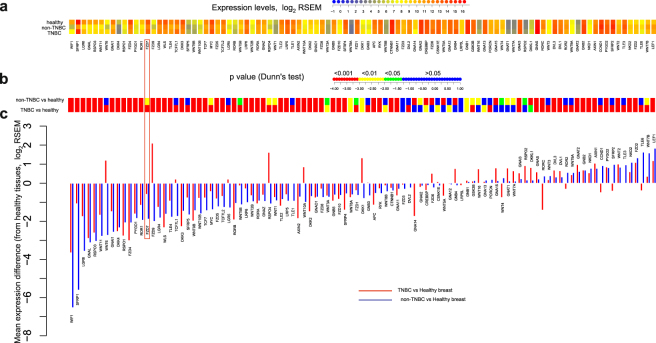
Expression levels of the Wnt pathway component genes in the TCGA tissue samples (113 healthy, 857 non-TNBC and 118 TNBC) (**a**) with p-values from Dunn’s multiple comparisons between them (**b**) and expression levels differences (relatively to healthy tissues) as a waterfall chart (**c**). FZD7 gene is highlighted on the figure (see manuscript text).

However, these findings suffer from the insufficient degree of consistency and ‘potency’. Lack of consistency is reflected by different proportions of BC patients revealing the change in expression pattern found in different reports (e.g. from 6% of patients with decreased APC expression in one study^22^ to 35% in another^21^). The lack of potency is illustrated by the fact that overexpression of e.g. FZD7 was only found to be two-fold in TNBC patients as compared with non-TNBC patients^11^ (FZD7 underexpression is actually seen in TNBC when compared to the healthy breast, see Fig. 1). Another shadow cast on these prior findings is that some of them were done with BC cell lines instead of patient samples; others relied on the promoter methylation analysis instead of measuring expression levels; and in some others, less reliable means of measuring expression levels were employed (i.e., microarrays or IHC quantification).

Network-based analysis provides an alternative to the investigation of changes in expression of individual selected genes^23–27^. Approaches of this type have demonstrated their potential in the search for novel drug targets and biomarkers, as exemplified by identification of 26S-proteasome genes as predictors of clinical outcomes in breast cancer^28^ or of the exploitation of phosphotyrosine signaling circuits in oncogenic transformation^29^. In addition to the identification of new players, this strategy can advance understanding of the mode of action of the already known oncogenes. For example, mutations in PIK3CA are well-described in many cancers, yet the Cancer Hallmark Network Framework^30^ analysis succeeded to identify a feed-forward loop of genes controlling PIK3CA activity and associated with worsened prognosis^31^. In the present work, we explore TCGA and GTEX gene expression data to exhaustively analyze Wnt pathway components in healthy and cancerous breast tissues. This network investigation derives correlation patterns and permits drawing important novel conclusions on the etiology and therapeutic approaches to BC.

## Results and Discussion

### Gene expression analysis confirms overall disinhibition of the Wnt pathway in BC but fails to identify specific targets

Analysis of expression levels of >100 Wnt pathway components (for the sources of the Wnt pathway component genes see Methods) in hundreds of healthy and BC samples provided by the RNA-seq data of the TCGA database^32^ fails to reveal striking changes in expression levels. Figure 1 summarizes these findings obtained for the healthy breast, non-TNBC and TNBC tumors, showing median gene expression (Fig. 1a) as well as statistical significance for its change compared to the healthy tissues (Dunn’s multiple comparisons) as heat-maps (Fig. 1b) or waterfall plots (Fig. 1c). Despite several components showing significant changes in the expression levels, these changes in most cases lie within the 2–4 fold interval (upregulated or downregulated) from the healthy breast levels (Fig. 1). Notable exceptions from this rule are WIF1 and sFRP1, the soluble inhibitors of the pathway, which show 10–50 fold drop in both non-TNBC and TNBC cancers as compared to control, agreeing with previous observations^19,33,34^.

These changes in expressions of the Wnt pathway components are translated into a consistent picture of the pathway overactivation emerging in BC as judged by the target genes’ expression levels (Supplementary Fig. 1). 34 genes in TNBC (and 23 in non-TNBC cancers) out of 138 known Wnt target genes show statistically significant >2-fold overexpression compared to healthy tissues (for the sources of the Wnt target genes see Methods). Some of the target genes, such as metalloproteinases MMP1, 9, and 3, show >10-fold overexpression in BC. Overall, these data support the picture of Wnt pathway overactivation in BC but suggest that maybe more subtle systemic changes underlie this dysregulation, rather than radical changes in expression of one or a few components, thus asking for a different approach to identify the potential drug targets.

### Strong correlations among gene expression levels of components of the Wnt and other signaling pathways are lost in BC

**Figure 2:**
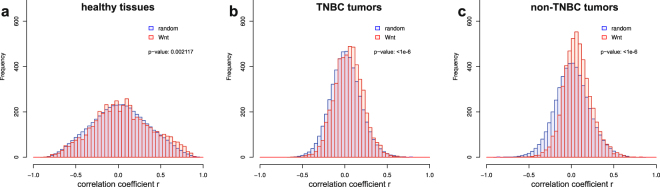
Distribution of the correlation coefficiets for the Wnt genes overlayed on average distribution of correlation coefficients in 500 random genes set in healthy tissues (**a**); TNBC (**b**) or non-TNBC (**c**) tumors. Pathway-related genes show significantly higher numbers of positive strong correlations as compared to the random genes in the healthy breast tissue, while gene co-regulation is dramatically lost in cancer both for random genes and Wnt pathway gene sets. The pathway distributions are narrower and more right-shifted as compared to average random set. P values are given from Kolmogorov-Smirnov test indicating unequality of distributions.

Failure to identify potential targets within the Wnt pathway by their expression levels prompted us to perform, instead of the simplistic single-gene expression analysis, a systems-level investigation of the Wnt pathway in healthy and BC tissues employing the correlation network analysis of gene expressions^23–27^. To this end, we computed all pairwise correlation coefficients between the expression levels of the genes encoding components of the Wnt pathway, both for BC samples and healthy controls. The results were compared to the similar analysis of 24 other KEGG signaling pathways, as well as to the randomly generated sets of genes (see Methods for details). As expected from prior works^35^, random sets of genes show high level of correlation of their expressions in the healthy tissues, which strongly drops in cancer tissues (Fig. 2). Genes encoding components of the Wnt pathway follow the same trend, but show considerably higher numbers of strong positive correlations in the healthy tissues (Fig. 2a), which is expected for the gene assemblies collectively regulating a physiologically important pathway functioning in a given tissue^36,37^. Interestingly, the similarly high correlation profile can be seen for the ERBB (HER2) pathway, known to be important in the breast and HER2-positive BC^38^, and to a lower extent – for the related Ras, Rap, PtdIns, and Sphingolipid pathways, but not so much for the other KEGG pathways (Supplementary Fig. 2).

If we take the strong positive and negative correlations (cut-off with the correlation coefficient │*r│* ≥ 0.5 and the p value ≤ 0.001) in the healthy breast analyzed over 113 patients, we find >800 correlations of the Wnt component genes, or close to 20% of total possible correlations. This number drops to 69 pairs in non-TNBC and 28 pairs in TNBC patients. Since the number of patient groups was unequal (850 in non-TNBC and 118 in TNBC), we randomly selected 5000 subsamples, 118 patients each, of the non-TNBC cohort and rerun the analysis, producing on average 86 ± 19 (mean ± SD) strongly correlating (│*r│* ≥ 0.5) pairs in the non-TNBC patients. These findings indicate that a hallmark of BC is the loss of coherence in expression (and hence activity) levels of the Wnt components observed in the healthy breast, potentially resulting in the dysbalancing of the Wnt pathway.

### Clusterization of the Wnt component genes provides clues to the Wnt-FZD interaction specificities

**Figure 3:**
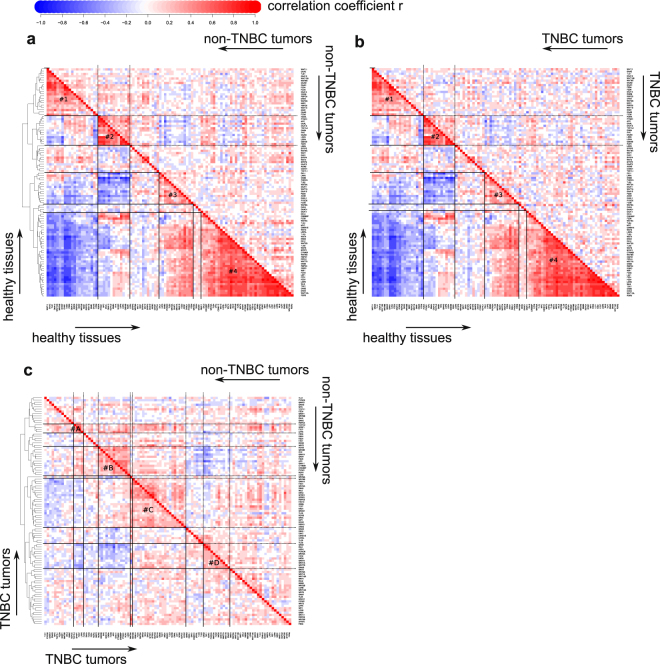
Comparison of the Wnt pathway components expression level correlations in healthy breast and breast cancer. Panel (**a**) shows clustering using the complete linkage algorithm for pairwise correlations in healthy breast (lower triangle of the heatmap) and for those observed in non-TNBC samples (upper triangle). The correlation clusters are highlighted by lines and numbered; the cluster #2 is the only one which can be observed in non-TNBC tumors (shown in dashed lines in the upper triangle). Panel (**b**) is similar to panel (a) but shows correlations in TNBC tumors in its upper triangle. Note that cluster #2 is also largely preserved. Panel (**c**) compares TNBC and non-TNBC tumors. Out of 4 gene clusters identified for Wnt component genes in TNBC, 3 (#A, #B and #C) are somewhat paralleled in non-TNBC (dashed lines).

Next, we applied the complete linkage method to clusterize the Wnt component genes within the healthy cohort, identifying four major clusters of the pathway components containing the genes highly correlating among themselves (Fig. 3a,b). The correlation clusters seen in the healthy tissue are essentially nonexistent in both non-TNBC and TNBC tumors with notable exception of cluster #2 (Fig. 3a,b, Supplementary Fig. 3a). The correlations remaining in BC can be independently clustered revealing four clusters (A to D) in TNBC (Fig. 3c and Supplementary Fig. 3d), component wiring in which is different from that of the healthy tissue with the exception of cluster #B containing most of the components of the healthy cluster #2 (Supplementary Fig. 3c,d). Comparative analysis of the TNBC and non-TNBC tumors (Fig. 3c and Supplementary Fig. 3e) reveals differences in the Wnt pathway organization between these BC subgroups. While clusters #A, #B and #C of TNBC can be partially seen in non-TNBC, cluster #D is lost. Independent clustering of non-TNBC identifies other major clusters within these tumors (Supplementary Fig. 3b,e).

Of the clusters identified in the healthy breast, cluster #4 is the biggest by the number of components (Fig. 3a). This cluster is positively associated with clusters #2 and #3 and is antagonistic to cluster #1. The existence of separate correlation clusters in the healthy breast cohort indicates that several subcomponents of the Wnt pathway are tightly co-regulated. Of the 19 Wnts and 10 FZDs, cluster #4 contains Wnt3, 4, 5a, 5b, 6, 7b, 8b, and 10a, which group together with FZD1, 3, 6, 7, and 10, while cluster #1 contains Wnt2, 2b, 9b, 10b, and 11, together with FZD4, 5, and 9 (Supplementary Fig. 3c).

This grouping provides an insight to the issue of the Wnt-FZD interaction specificities. The promiscuity of these ligand-receptor interactions, coupled with partial selectivity, has been a mystery for the Wnt field for decades. Many systematic attempts to categorize these interactions have been applied, relying on different, largely biochemical, approaches (reviewed in^39^). However, it is clear that the ability of a given Wnt to interact with a given FZD, measured e.g. in the binding assay, does not necessarily translate into the functional activation of the intracellular signaling, nor proper physiological response in the context of a given tissue. Our network correlation analysis provides an interesting alternative to these prior attempts to comprehend the ligand-receptor specificity/promiscuity in the Wnt pathway. We propose that, at least in the context of the healthy breast tissue, physiologically relevant and distinct large subgroups of Wnt/FZD interactions can be identified (Fig. 3 and Supplementary Fig. 3). Within each subgroup, promiscuity dominates, but strict antagonistic selectivity is found between these subgroups. Curiously, the so-called canonical Wnts are found to functionally group together with the non-canonical Wnts (e.g. Wnt3 together with Wnt5a in cluster #4), indicating that the canonical *vs*. non-canonical distinction does not reflect the co-expression pattern and may therefore not be strongly physiological relevant in the current context.

### Correlation network analysis validates the breast Wnt target genes

**Figure 4:**
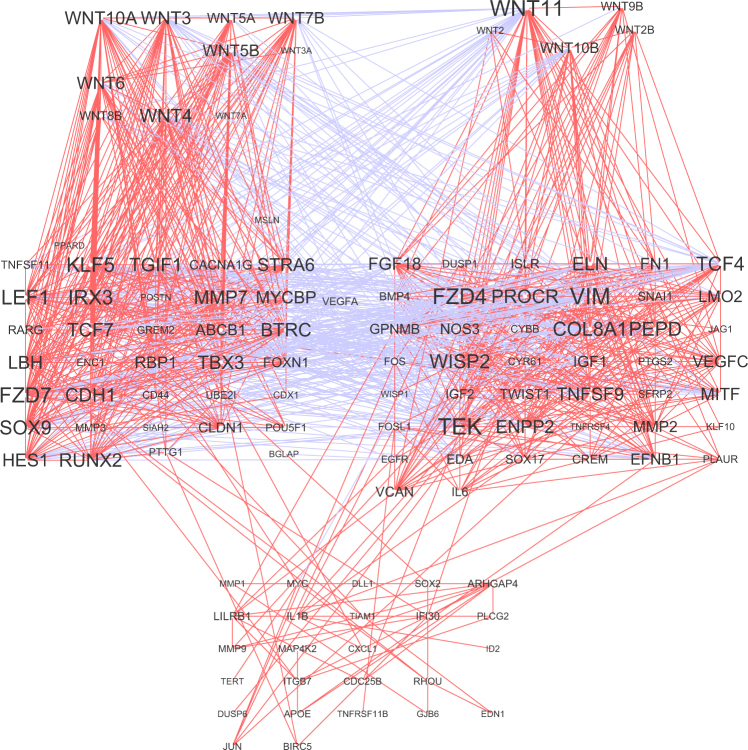
Network showing dependency of different Wnt target genes expression levels on Wnt ligands. Target genes can be separated in two essentially antagonistic clusters controlled by Wnt ligands from clusters #4 (left) and #1 (right) as shown on Fig. 3. However, there is also a low-connected cluster of target genes which seems to be correlating with target genes of both clusters.

We hypothesize that Wnt-FZD interactions permitted within the different pathway component clusters initiate different subtypes of the Wnt signaling in the healthy breast tissue. This hypothesis is supported by the ability of different component clusters to control separate pools of target genes (Fig. 4). Further, this analysis permits validating the Wnt target genes in regard to a particular tissue, in this case the breast, as the tissue specificity of the Wnt targets has been challenging to explain for a long time and was mostly studied using artificial systems^40,41^. Our analysis provides a list of target genes, which strongly correlate with Wnts of the two main clusters (#4 and #1) and are therefore likely to be the relevant Wnt pathway target genes in the breast (Fig. 4 and Supplementary Table 1). Surprisingly, some of the most “popular” target genes such as Myc, LGR5 and AXIN2 known to depend on Wnt signaling in other tissues do not show up among the most relevant players in the healthy breast tissue (Supplementary Table 1).

### Patient stratification based on Wnt signaling in TNBC

**Figure 5:**
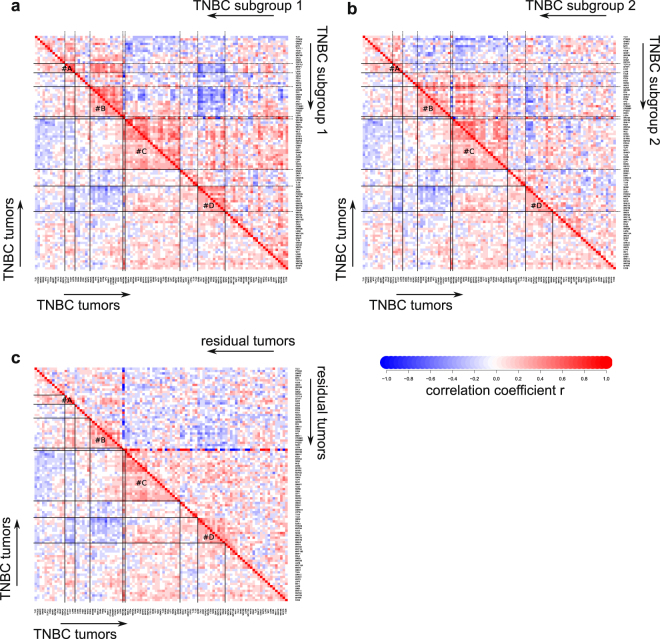
Heatmaps comparing initial set of TNBC tumors with subgroups obtained upon search for subgroups with increased number of correlation. Panel (a) compares subgroup 1, panel (b) subgroup 2 and panel (c) shows tumors which do not belong to any subgroup (“residual tumors”). Most of the clusters identified in original TNBC set remain and show increased component “wiring”.

The drop in correlations and their re-wiring as compared to the healthy tissues indicate the significance of the Wnt pathway dysregulation in BC. We wondered whether the low number of correlations left in BC could partially be due to existence of distinct patient subgroups, each characterized by their own correlations, which disappear through superposition when these subgroups are combined together. To test this hypothesis, we designed an algorithm to stratify the TNBC patients into subgroups with maximal internal quantity of correlations among the Wnt component genes (see Methods for technical details on the subgroup selection), revealing two subgroups (38 and 39 patients each) with 6- to 10-fold higher numbers of strong correlations (Fig. 5a,b). The Wnt pathway network appears wired differently in these subgroups (Supplementary Fig. 4a–c). “Residual” tumors show randomization of the cluster relationships (Supplementary Fig. 4d) and may have found their own way of the Wnt pathway exploitation or do not rely on it at all. In agreement with the network correlation analysis, we find that the TNBC subgroups are characterized by significant differential expression (some reaching 4–6 folds) of many Wnt pathway components (Supplementary Fig. 5).

We suggest that the TNBC subgroups we have identified will have common vulnerability and therefore should respond differently to the anti-Wnt treatments (eventually to be developed) tailored against their corresponding essential components. Supplementary Table 2 lists the Wnt component genes with the higher degree of connectivity belonging to the two TNBC stratifications. Among them, the strongest (by the correlation coefficient r) pairwise correlations (Supplementary Table 3 and Supplementary Fig. 6) may indicate proteins with concerted action within the pathway and/or with physical interactions. Some of them appear to be shared between the subgroups while others can be unique for one of the two subgroups. Interestingly, the highest diversity is observed among the Wnts and FZDs with only Wnt7a being shared. As examples of highly correlating pairs acting at the higher ‘floors’ of the Wnt pathway, we mention WNT9B-FZD4, FZD7-LRP6, FZD4-GNA14, FZD2-GNAI2 and RORA-GNA13 (complete TNBC cohort); WNT5B-ROR2, FZD6-GNA13, FZD2-GNAI2, ROR2-GNAI2, and GNB2-DVL1 (TNBC subgroup 1); and FZD4-GNA14, RORA-GNA14, RORA-GNA13 (subgroup 2) (Supplementary Data Table 3 and Supplementary Fig. 6). Several of these interactions involve α- or β-subunits of the heterotrimeric G proteins. The importance of the G proteins in Wnt/FZD signaling has been shown in a number of studies (reviewed in^39,42,43^), but their potential clinical importance in the Wnt pathway has not emerged previously. Future investigations will show whether specific targeting of these interacting pairs may indeed provide the clinical significance combined with the required drug selectivity.

**Figure 6:**
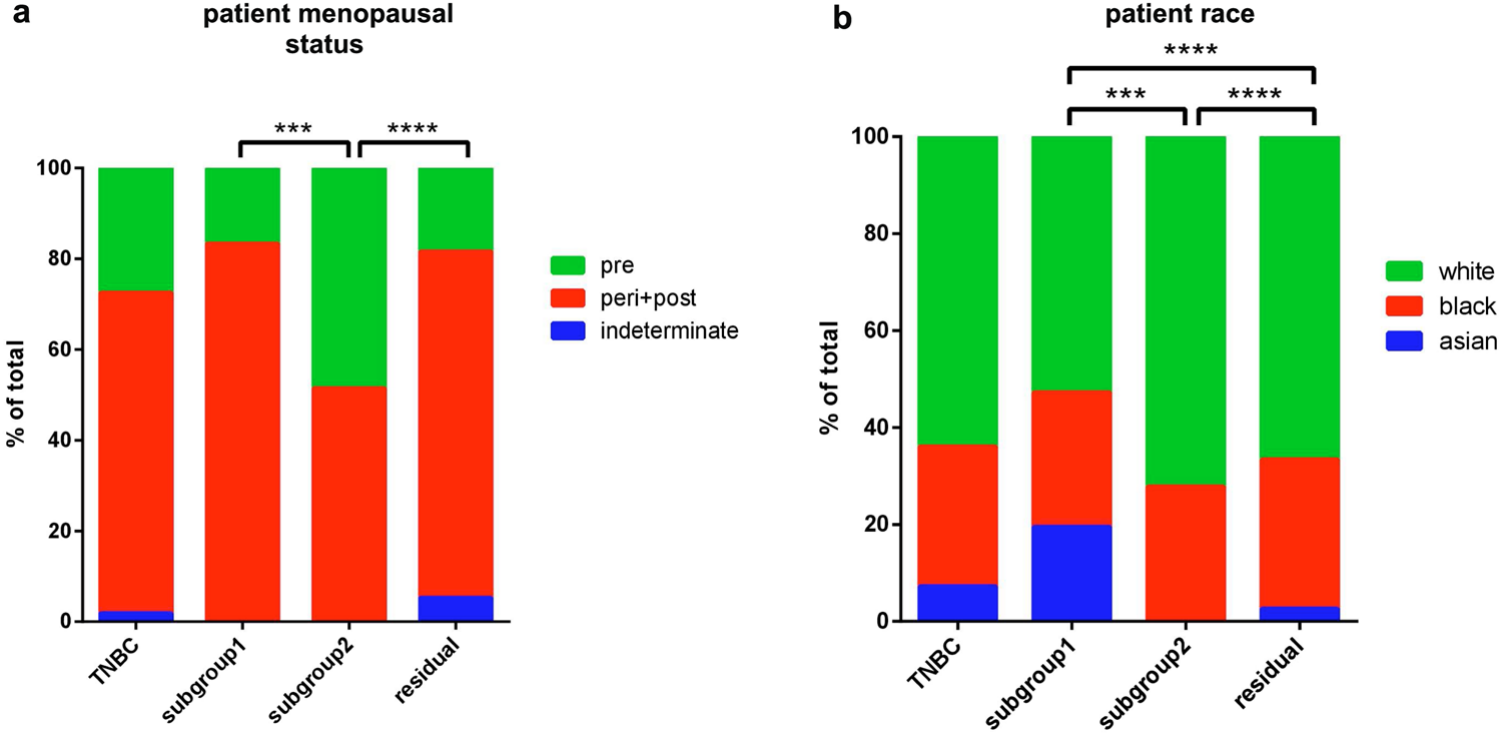
Analysis of clinical features of patients belonging to TNBC subgroups. Panel (**a**) shows that subgroup 2, as compared to subgroup 1 and “residual” tumors, contains an increased proportion of pre-menopausal women. (**b**) shows distribution of race of the patients belonging to the different subgroups, with clear enrichment of Asian patients in subgroup 1 and their decrease in combination with increase of white patients in subgroup 2. Statistical significance was estimated by chi-squared test.

We assessed 110 clinical characteristics of the patients available in TCGA to see if they can be associated with the TNBC subgroups identified, finding the patient race and menopausal status to be a distinguishing factor (Fig. 6). Subgroup 1 shows significant propensity to occur more frequently in post and peri-menopausal (Fig. 6a) and Asian patients (Fig. 6b), while subgroup 2 contains more white patients and shows propensity to include pre-menopausal women. Other clinical factors, such as e.g. the clinical score of the tumor (Supplementary Fig. 7), also reveal certain degree of association with the TNBC subgroups.

### Comparative analysis of the TCGA and GTEX datasets highlights genes with potential roles in the cancer-stroma interaction

We complemented our network correlation analysis performed with the TCGA data by the RNAseq data obtained from non-cancer patients available at the GTEX database^44^. When performing the network correlation analysis on breast GTEX samples, we find a strong similarity with the TCGA data reflected by the preservation of Wnt pathway clusters #2 and #4. At the same time, we see a decrease in the connectivity within cluster #1, although their strong antagonistic interactions with cluster #4 remain (Supplementary Fig. 8). Although the exact reason(s) for these changes is unclear, the healthy tissue samples of the TCGA database, being taken 2–3 cm from the tumor site, may have been affected by the neighboring tumor. To highlight the most significant differences between the TCGA and GTEX data, we calculated the Δr difference observed for the same Wnt component pairs in the two datasets and ranked the genes by the quantity of their correlations changing with Δr > 0.5. Wnt10B stands at the top of this rank with 58 correlations being strongly changed (and essentially inverted) (Supplementary Fig. 9). Wnt10B may thus emerge as an important player in the Wnt pathway rewiring occurring in the BC microenvironment.

### Independence of the strong correlations from tissue heterogeneity

We were concerned whether cell type heterogeneity of the healthy and cancerous breast tissues could influence the correlations we observed. At least two cell types (adipose and ductal epithelial cells) are present at similar proportions in any healthy breast sample; stromal and immune cells will also ‘contaminate’ the tumor. If one gene is stably expressed in one cell type, and another gene – in another cell type, a cohort of tissue samples with varying levels of heterogeneity in the proportions of these two cell types will produce a false correlation in expression of these two genes. To address the question of how many correlations reported in this paper might be such false correlations, we created a stochastic model comprising a set of patient samples composed of two cell types in different proportions, with different gene expression levels in these cell types (Supplementary Fig. 10). The actual median expression levels of the Wnt pathway component genes, along with their standard deviations available from GTEX for 53 different tissues, were used in the model to randomly assign to the two cell types for 118 patients. Additionally, each of these patients was assigned a value of the proportion of these two cell types, varying either the mean sample heterogeneity (keeping the distribution constant, Supplementary Fig. 10a) or the distribution of the sample heterogeneity (keeping the mean heterogeneity constant, Supplementary Fig. 10d). These different conditions were used next to calculate the resulting gene expression pattern for the Wnt pathway component genes. Means of the distributions of the correlation coefficients resulting from 500 such simulations are shown on Supplementary Fig. 10b,c,e,f (with or without standard deviations) superimposed on the actual distributions of the correlation coefficients observed in the healthy or cancerous tissues (see Fig. 2). This simulation reveals that the actual mean level of tissue heterogeneity has a little effect on the likelihood of observing highly correlating genes (Supplementary Fig. 10b,c). A higher influence is exerted by the distribution of the heterogeneity across the samples, such that cohorts, some samples of which have close to 100% content of only one or only the other cell type, may result in high amounts of false strong correlations (red bars and lines in Supplementary Fig. 10d–f). However, such distribution of the cell type heterogeneity in the bioptates is impossible and contradicts statements on the sample quality control from TCGA and GTEX as well as works estimating purity of the samples in TCGA^32,44,45^. Our modeling thus confirms that the correlations we observe are real and result from real co-regulation of the gene expression in the breast.

Overall, our work reveals an unanticipated system of checks and balances ensuring the properly regulated functioning of a key signaling pathway in the healthy breast. Dramatic loss of this system, likely through epigenetic dysregulation, unleashes uncontrollable Wnt pathway activation causing oncogenic transformation, to a high degree individualized in each patient. Our work also opens new avenues for targeted drug discovery in BC.

## Methods

TCGA datasets with pre-calculated RSEM for RNA-seq quantification of gene expression, normalized RNA chip data and patient’s clinical data have been downloaded from Broad institute Firebrowse website (http://firebrowse.org, cohort BRCA (Broad Institute TCGA Genome Data Analysis Center (2016): Firehose 2016_01_28 run. Broad Institute of MIT and Harvard. doi:10.7908/C11G0KM9). Most recent version of GTEX RNA-seq database (v6) has been downloaded from GTEX website^44^.

The gene list for the core 101 Wnt component genes (and all their orthologs) (Supplementary Table 1) has been compiled based on the data available in reviews^43,46^; additionally it was merged with the complete list of G protein α, β, and γ subunit orthologs, given the importance of heterotrimeric G proteins in Wnt signaling (reviewed in^39,42,43^). The list of 138 validated Wnt target genes was assembled using information provided on website of Nusse laboratory (http://web.stanford.edu/group/nusselab/cgi-bin/wnt/), NetPath^47^ and literature^4^. Several Wnt component genes are also well-proven target genes of the pathway, such as FZD^7^, FZD^4^, WNT3A^4,48,49^.

TCGA BRCA patients were separated in two cohorts by their clinically reported receptor status: non-TNBC (852): ER+/PR+ and HER+ (or both) and TNBC (118) with the negative status for all three receptors. Patients with healthy breast tissue analyzed (113) were treated as a single cohort, though tumors of 12 of them belonged to the TNBC cohort and the others had non-TNBC tumors.

We have confirmed the non-mutational and non-genetic nature of the Wnt pathway modulation in BC, using the CBioPortal interface^50^, which provides genomic data analysis for the tumors available in TCGA. For each of the 101 Wnt component genes, the mutation rates in BC rarely exceeded 1% (most of them <0.2%) (Supplementary Fig. 11). There are chromosomal rearrangements affecting Wnt component genes in a significant number of BC patients (1–10%, Supplementary Fig. 11), however as it is clear from the data on the Fig. 1, they do not lead to or correlate with strong gene expression changes.

Tables containing log levels of RSEM (for TCGA RNA-seq data), RPKM (GTEX data) or chip median expression levels (for TCGA RNA chip data) for component or target genes were used to build corresponding figures using scripts developed in R^51^ (using packages Hmisc^52^ and gplots^53^) and export pairwise correlation data in Cytoscape^54^ for building the networks. The complete linkage algorithm in the built-in R function *hclust* has been used to create dendrogram and identify major clusters of the genes in all cases.

To confirm the validity of the correlation networks we have identified, Supplementary Fig. 8a shows comparison of the correlations obtained for the same TCGA samples using RNA chip with those from RNAseq. With a two-fold lower number of samples, the RNA chip picture is essentially “low contrast” of the RNA-seq data, indicating both superiority of the RNA-seq in precision of RNA levels estimate and basic independence of the correlations and key components from the exact method applied.

For search of the subgroups within TNBC cohort, the following algorithm was implemented: the patients were grouped in blocks of 5. For all possible groups formed by combinations of five such blocks (53130) a number of strong (|r| > 0.5) and highly significant (p < 0.001) correlations was calculated. The five blocks of patients most frequently appearing in the 100 top-scoring combinations were picked. For this group (containing 107 strong correlations over 28 in original TNBC cohort), enriched in patients belonging to a certain subgroup, we removed the “wrong” patients by iteratively deleting two patients, removal of which resulted in the maximal gain of correlations until the removal of the next pair resulted in decreased or unchanged number of correlation. The group containing the maximal number of correlations was used as a “core group” to extract the patients belonging to the subgroup from the remainder using the inverse approach: two random patients were iteratively added to the “core group” and those, which provided the maximal increase in the number of strong correlations, were added. These iterations continued until the number of correlations started to decrease with addition of the next pair. The last pair was considered a cut-off for the first subgroup (containing 246 strong correlations) and the remainder of the patients was treated the same way resulting in identification of the second subgroup (133 correlations). Finally, the “residual” group was treated the same way, however it did not contain any combinations with significantly increased number of correlations over the parental subgroup (fluctuating within a window of 30–40 strong correlations) and therefore was considered to not contain any smaller subgroups.

The R scripts and data tables which has been used to produce the figures and for TNBC subgrouping are available in corresponding section on the public website of Katanaev lab (https://www.unil.ch/dpt/en/home/menuinst/recherche/groupe-katanaev/files.html).

## Electronic supplementary material

Supplementary figures and tables
