# Supplementary material for: Dramatic dysbalancing of the Wnt pathway in breast cancers
Source: Sci Rep. 2018 May 9;8:7329. doi: 10.1038/s41598-018-25672-6 (PMC5943245; doi:10.1038/s41598-018-25672-6)
Supplement: Supplementary file 1 — Supplementary figures and tables [file 41598_2018_25672_MOESM1_ESM.pdf]

Supplementary information for:

Dramatic dysbalancing of the Wnt pathway in breast cancers

Alexey Koval[1] and Vladimir L. Katanaev[1,2]*

[1]Department of Pharmacology and Toxicology, Faculty of Biology and Medicine, University of Lausanne, Lausanne 1011, Switzerland.

[2]School of Biomedicine, Far Eastern Federal University, Vladivostok, Russian Federation.

[*]to whom correspondence should be addressed: Vladimir.katanaev@unil.ch; Tel. +41 21 692 5459.

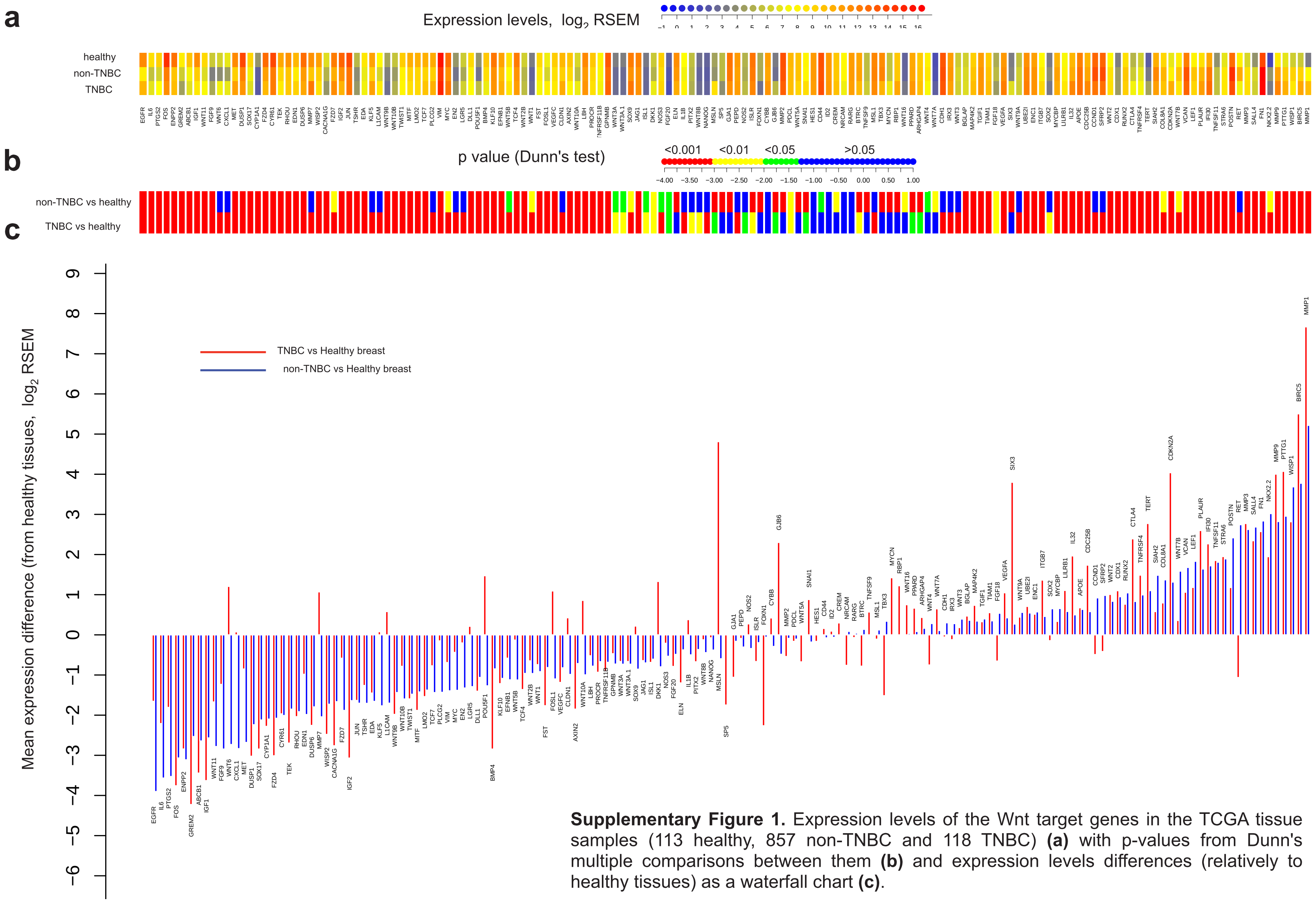

Supplementary Figure 1. Expression levels of the Wnt target genes in the TCGA tissue samples (113 healthy, 857 non-TNBC and 118 TNBC) **(a)** with p-values from Dunn's multiple comparisons between them **(b)** and expression levels differences (relatively to healthy tissues) as a waterfall chart **(c)**.

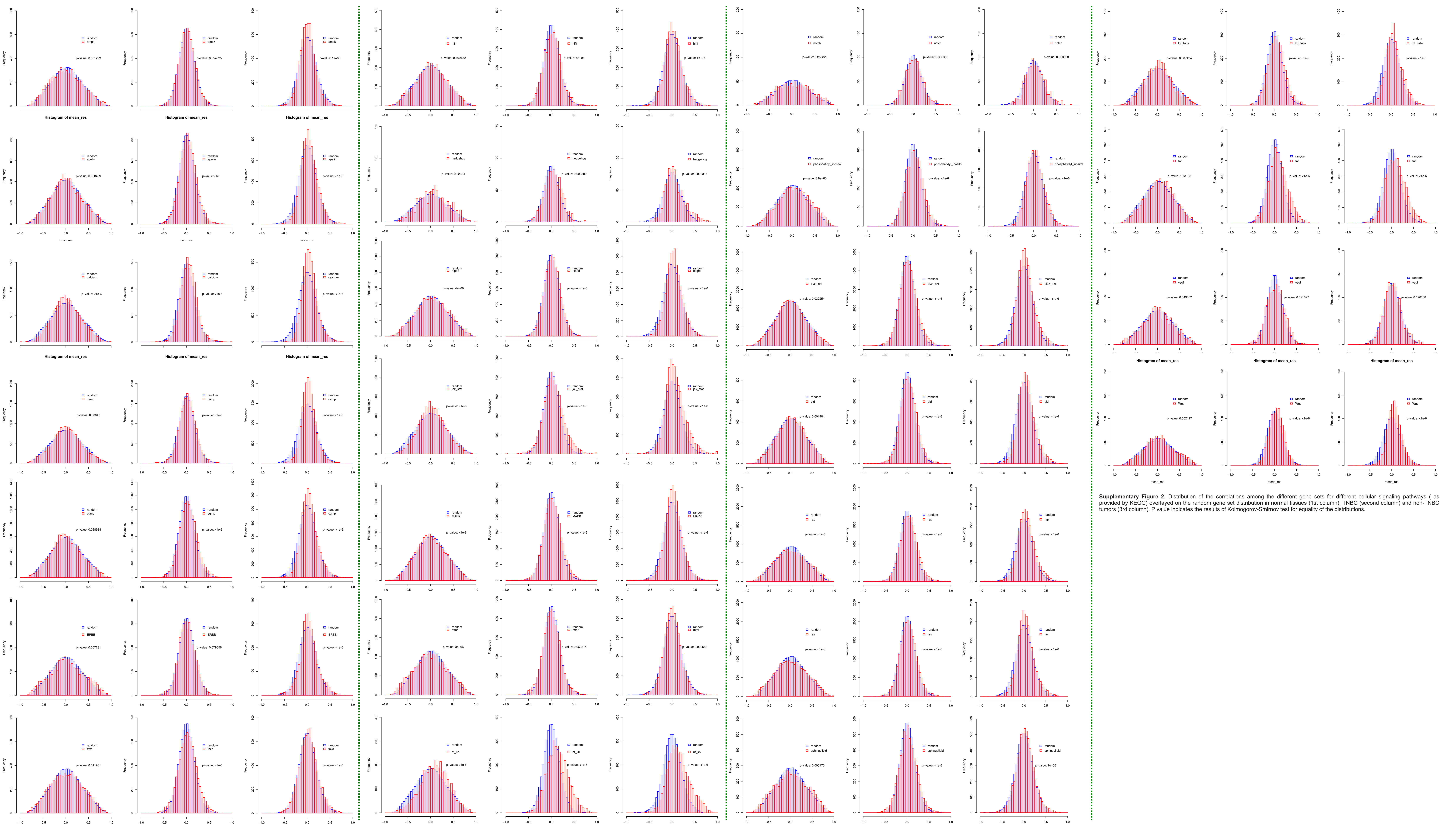

Supplementary Figure 2. Distribution of the correlations among the different gene sets for different cellular signaling pathways (as provided by KEGG) overlayed on the random gene set distribution in normal tissues (1st column), TNBC (second column) and non-TNBC tumors (3rd column). P value indicates the results of Kolmogorov-Smirnov test for equality of the distributions.

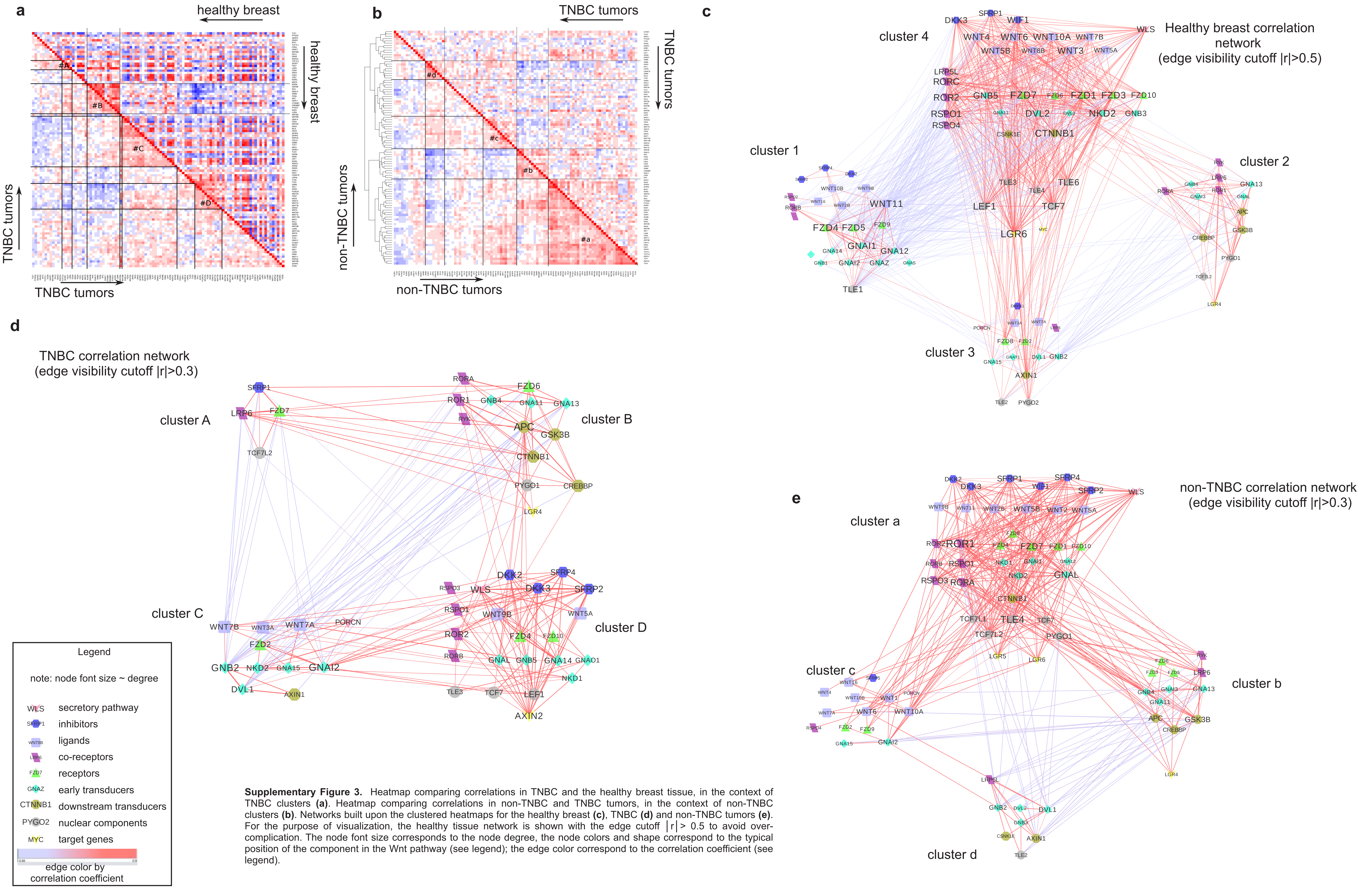

Supplementary Figure 3. Heatmap comparing correlations in TNBC and the healthy breast tissue, in the context of TNBC clusters **(a)**. Heatmap comparing correlations in non-TNBC and TNBC tumors, in the context of non-TNBC clusters **(b)**. Networks built upon the clustered heatmaps for the healthy breast **(c)**, TNBC **(d)** and non-TNBC tumors **(e)**. For the purpose of visualization, the healthy tissue network is shown with the edge cutoff |r|> 0.5 to avoid over-complication. The node font size corresponds to the node degree, the node colors and shape correspond to the typical position of the component in the Wnt pathway (see legend); the edge color correspond to the correlation coefficient (see legend).

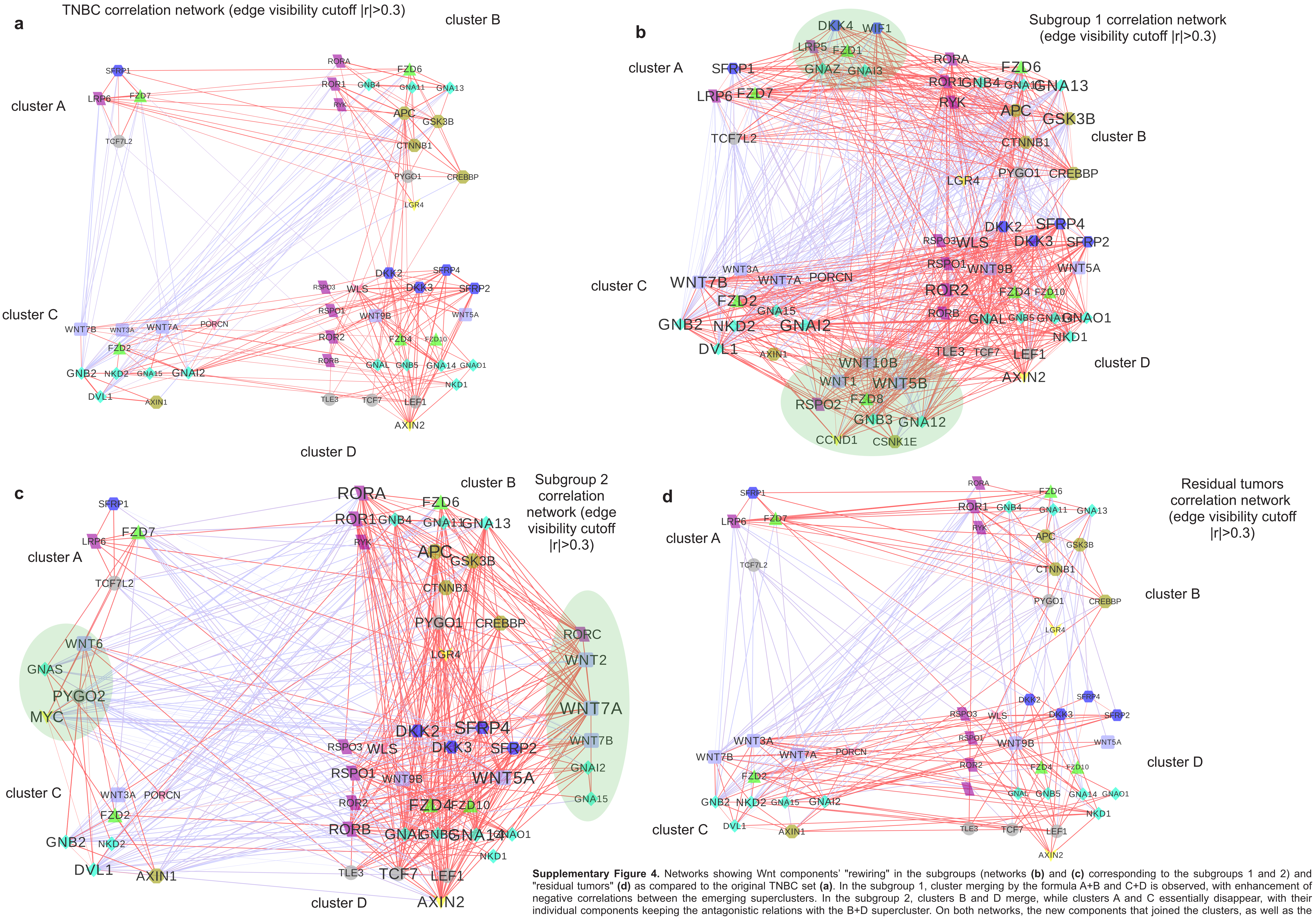

Supplementary Figure 4. Networks showing Wnt components' "rewiring" in the subgroups (networks **(b)** and **(c)** corresponding to the subgroups 1 and 2) and "residual tumors" **(d)** as compared to the original TNBC set **(a)**. In the subgroup 1, cluster merging by the formula A+B and C+D is observed, with enhancement of negative correlations between the emerging superclusters. In the subgroup 2, clusters B and D merge, while clusters A and C essentially disappear, with their individual components keeping the antagonistic relations with the B+D supercluster. On both networks, the new components that joined the clusters, as well as the components that strikingly changed their "wiring" are grouped and highlighted in green.

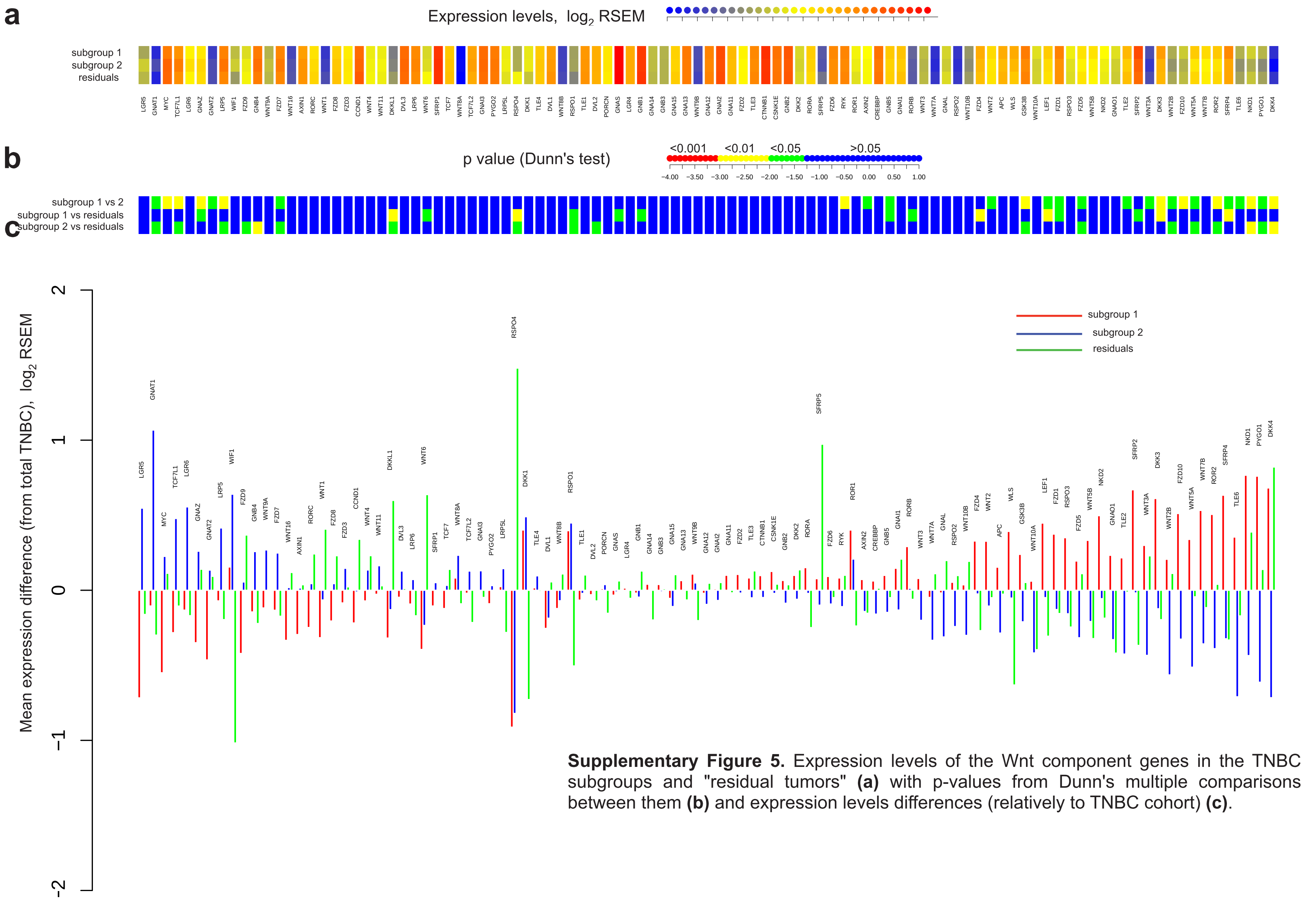

Supplementary Figure 5. Expression levels of the Wnt component genes in the TNBC subgroups and "residual tumors" **(a)** with p-values from Dunn's multiple comparisons between them **(b)** and expression levels differences (relatively to TNBC cohort) **(c)**.

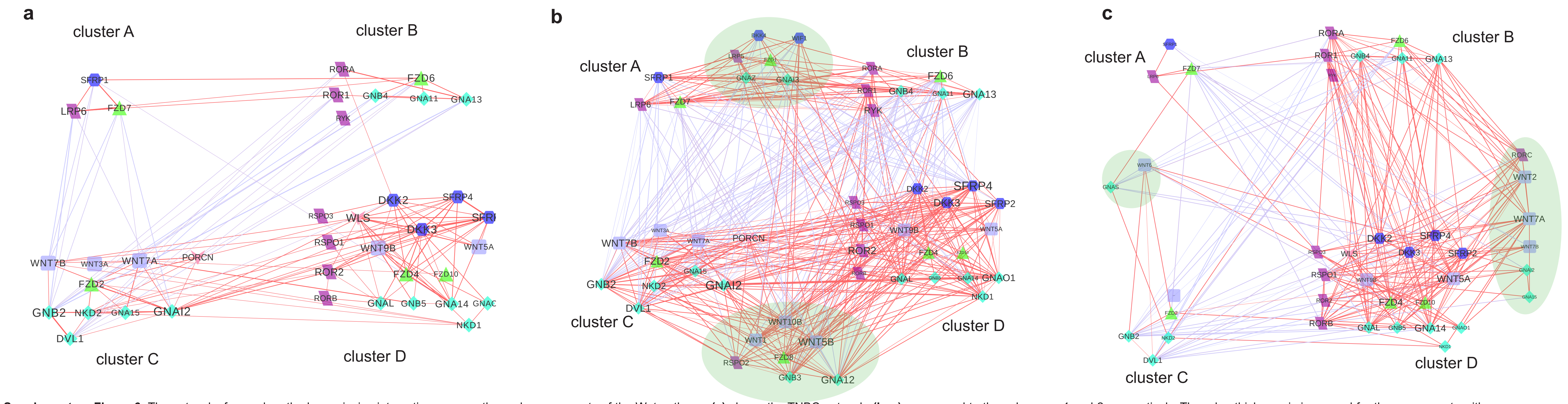

Supplementary Figure 6. The networks focused on the key pairwise interactions among the early components of the Wnt pathway. **(a)** shows the TNBC network. **(b, c)** correspond to the subgroups 1 and 2, respectively. The edge thickness is increased for the components with the high correlation coefficient. The font size is increased for the key nodes with the high degree (calculated from the parental network of Supplementary Figure 2). Some of these interactions might be physical (see Supplementary Table 3), revealing potential drug targets for the specific therapies.

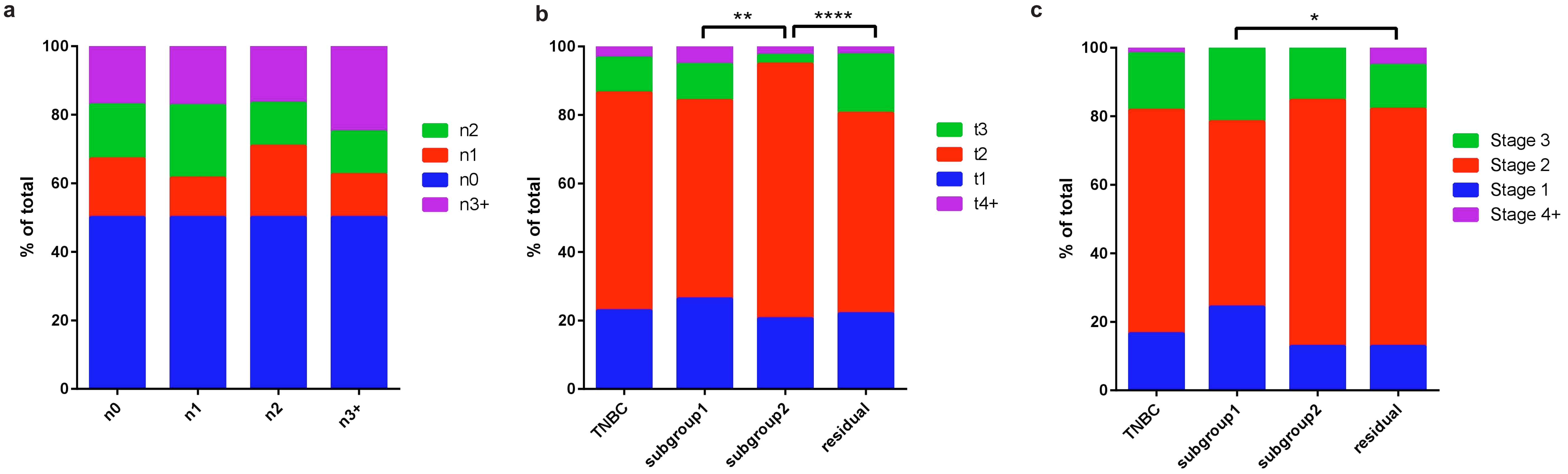

Supplementary Figure 7. Analysis of clinical features of patients belonging to TNBC subgroups. Panels show proportions of patients' tumor n- **(a)** and t-scores **(b)**, as well as the tumor stage **(c)** within the subgroups and the initial TNBC cohort. Statistical significance was assessed by the chi-squared test.

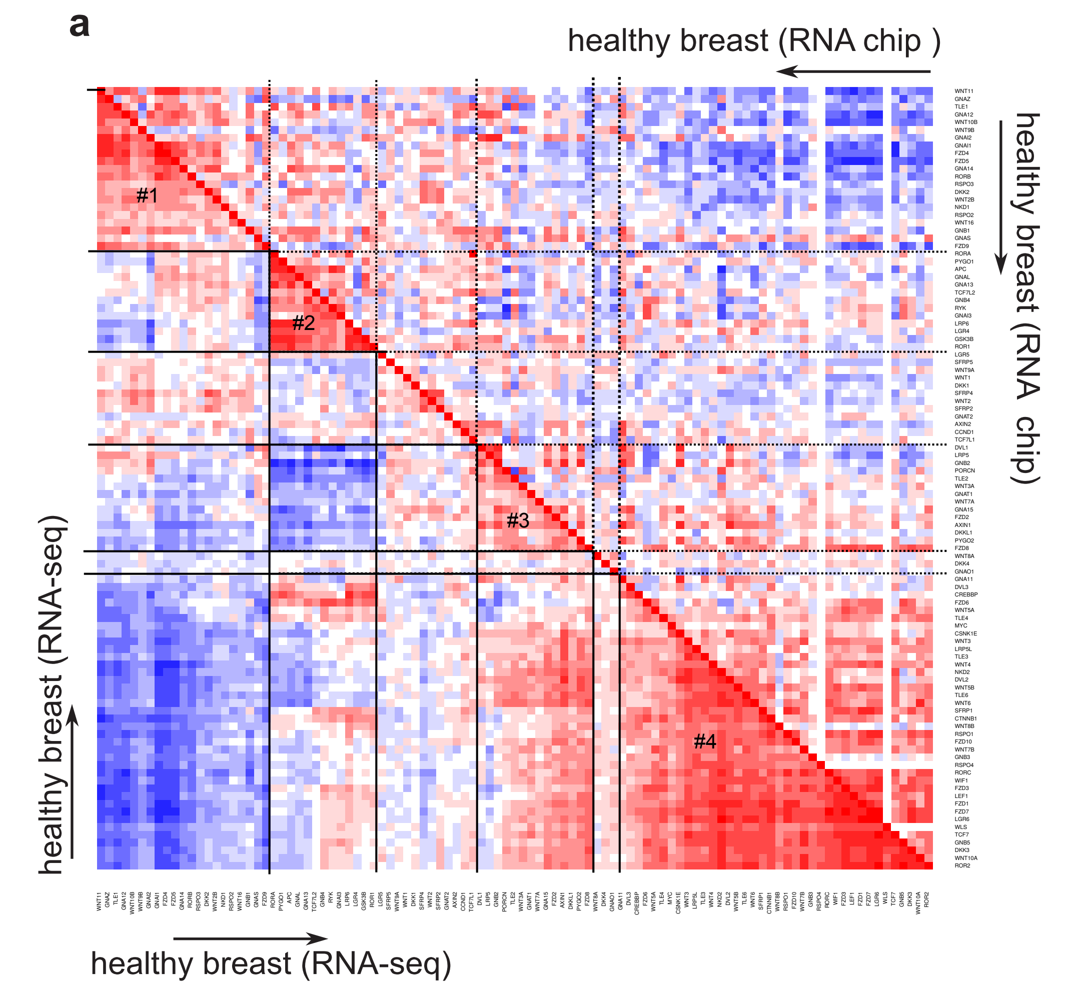

Supplementary Figure 8. (a) Heatmap comparing pairwise correlations of Wnt component genes in healthy tissues obtained by RNA-seq with those obtained in parallel by RNA chip quantification (both from TCGA). **(b)** Heatmap comparing pairwise correlation of Wnt component genes in healthy breast tissues from TCGA with those for healthy female breast tissues in GTEX. **(c)** Heatmap comparing pairwise correlation of Wnt component genes in healthy breast tissues from TCGA computed using Pearson against those calculated using the Spearman method.

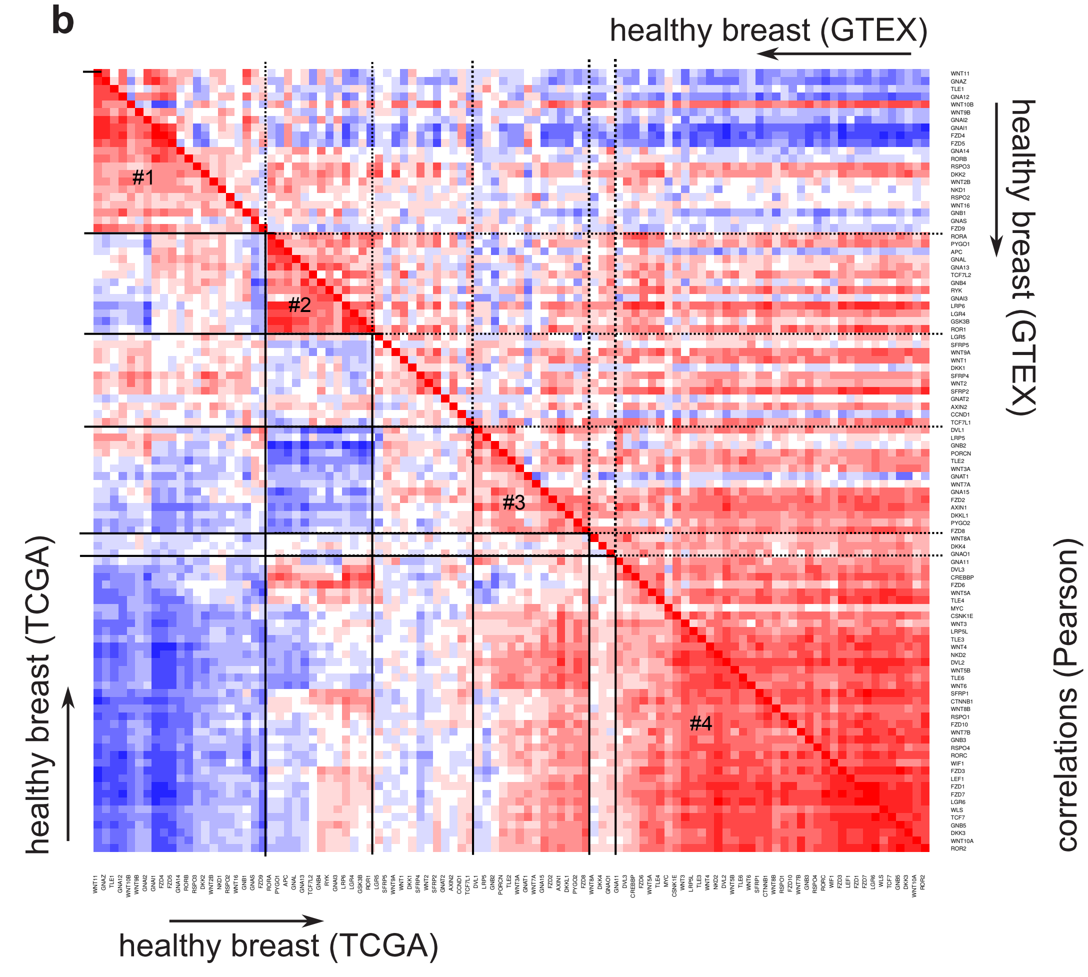

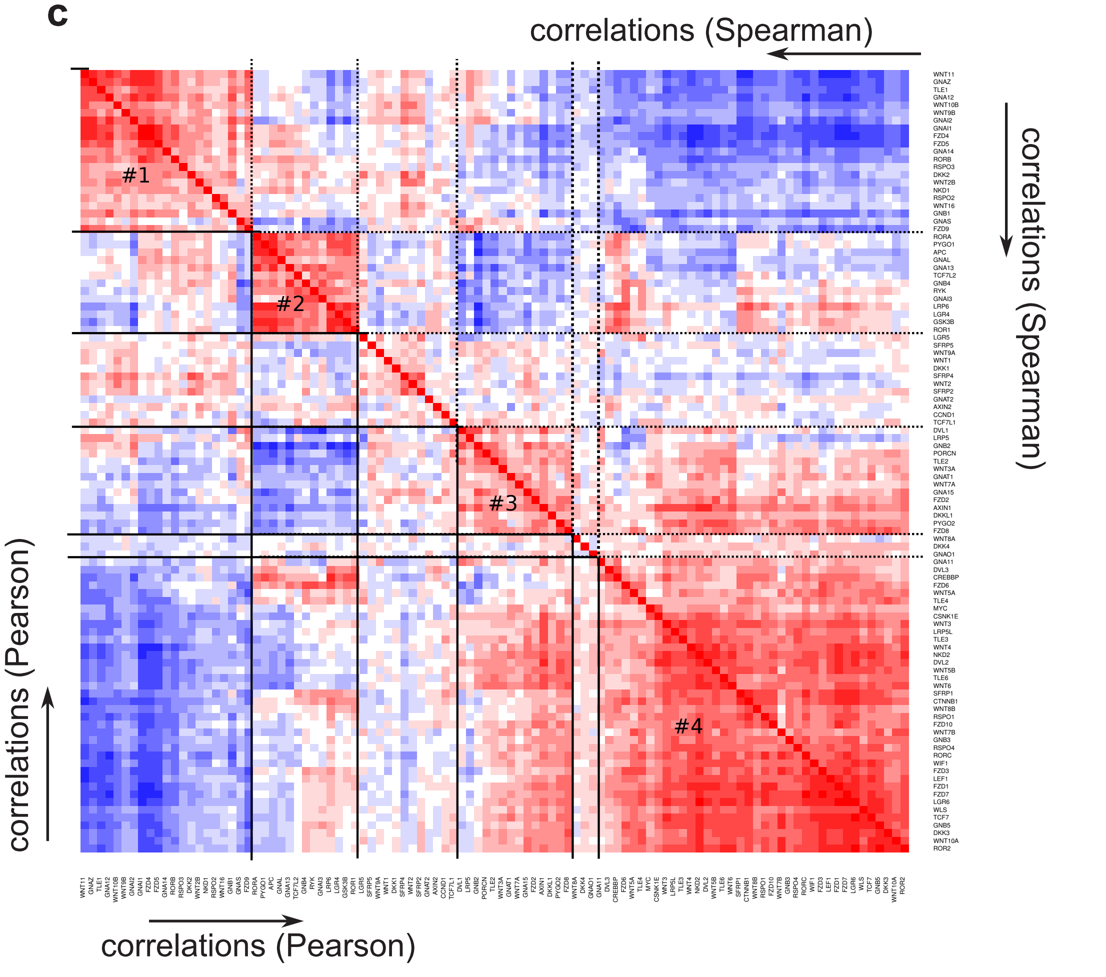

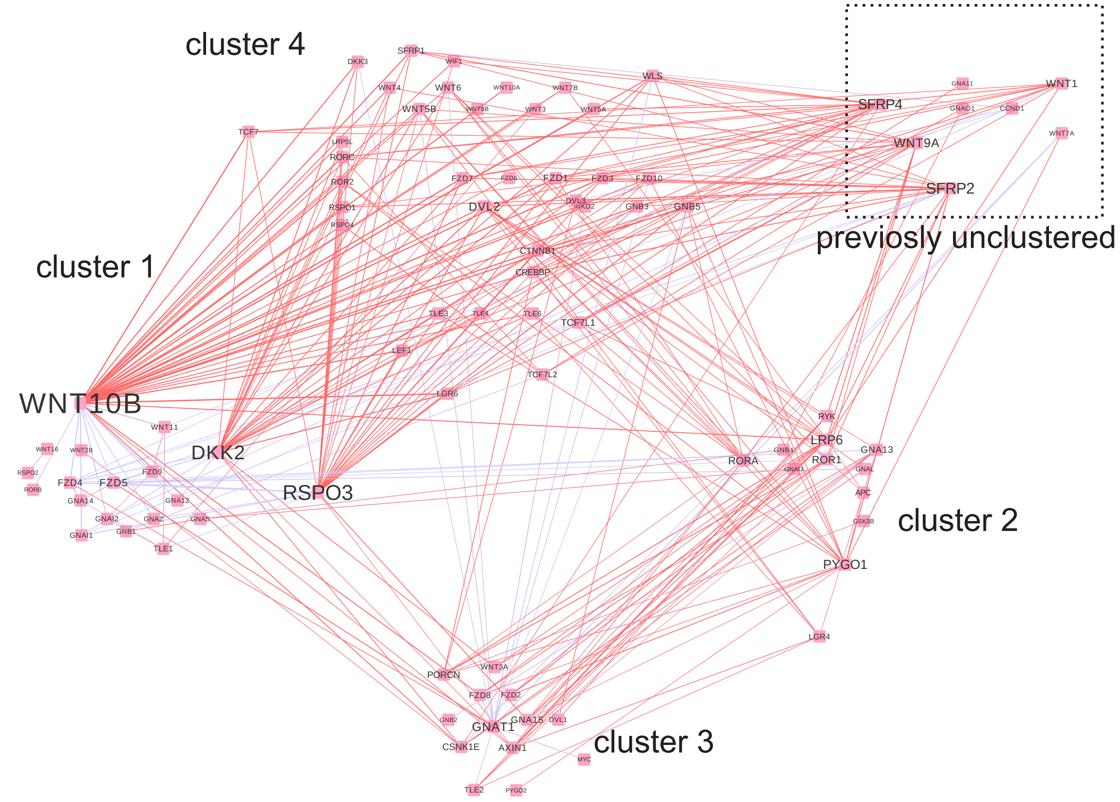

Supplementary Figure 9. Network of the genes showing significant changes in correlation coefficient (Δr) between TCGA and GTEX. Genes are clustered as previously for TCGA, edge colour corresponds to the correlation coefficient observed in GTEX (as for the Supplementary Figure 2), edge thickness is proportional to Δr.

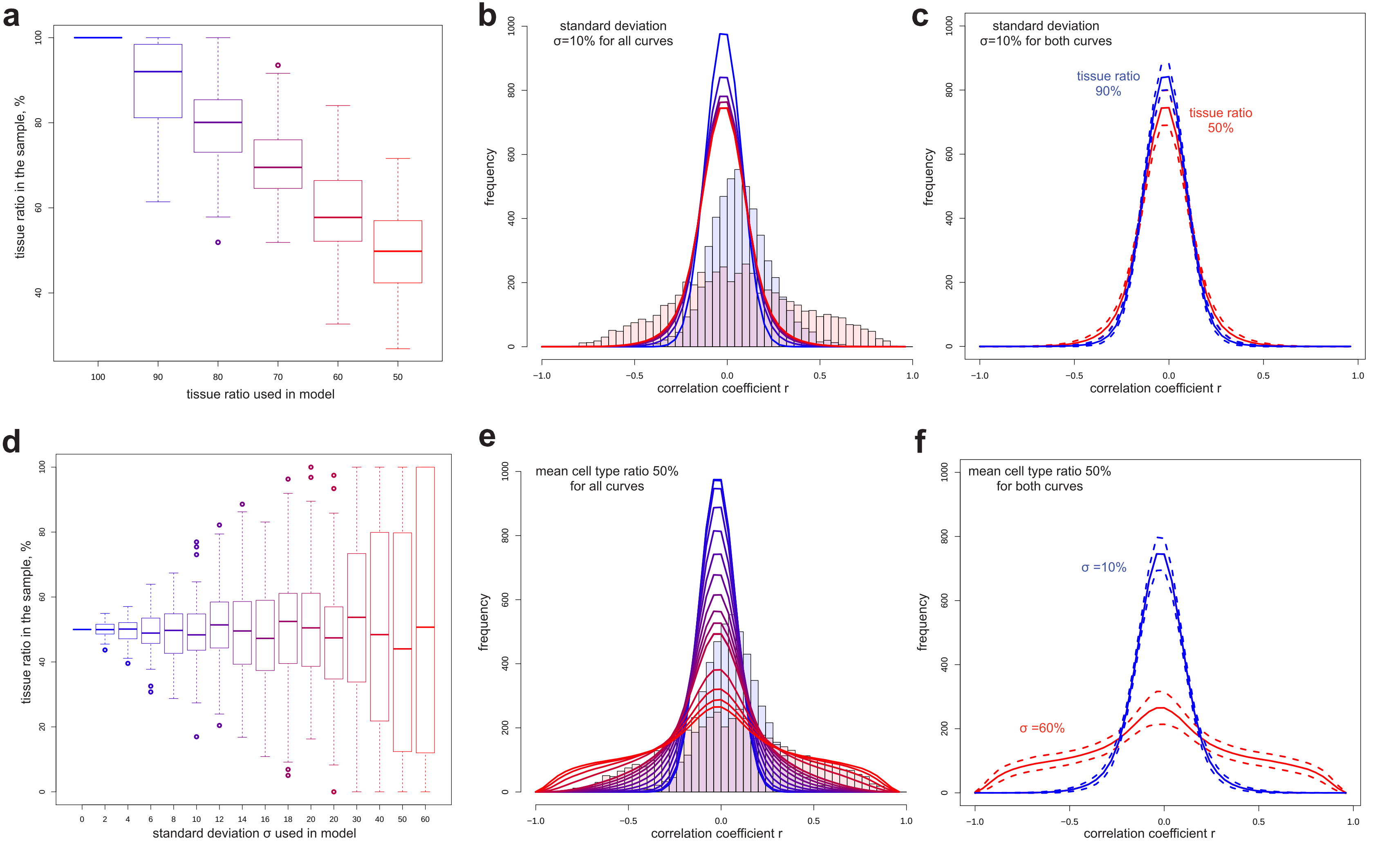

Supplementary Figure 10. Mathematical modeling of correlation patterns in the presence of two cell types reveals that tissue purity variance, rather than the mean tissue purity, may influence the resulting gene expression correlations. Box plots show distribution of modelled sample purity, with **(a)** keeping the distribution constant and varying the mean purity, and with **(d)** fixing the tissue purity at 50% and increasing the deviation in the tissue purity. The charts shown in **(b)** and **(e)** illustrate the average distributions of correlation coefficients (curves) obtained for samples from (a) and (d) (each curve is the mean of 500 simulations) superimposed on the real distributions (bars) observed in the TNBC and healthy tissues (see Fig. 2). The charts shown in **(c)** and **(f)** illustrate selected examples from the curves from (b) and (e), respectively, with standard deviations provided as dashed lines. The sample size for each simulation is 118 tumors. The gene expression parameters for the Wnt pathway components are taken for the modelling from the GTEX database of healthy tissues (see Methods for the detailed description of the modelling parameters).

Supplementary Figure 11. Oncoprint report from CBioPortal for the set of Wnt component genes in entire BC population. In multiple patients, some genes are affected by chromosomal rearrangements (amplifications or deletions, see legend below); mutations are also seen in some cases. However, these changes are rather individual.

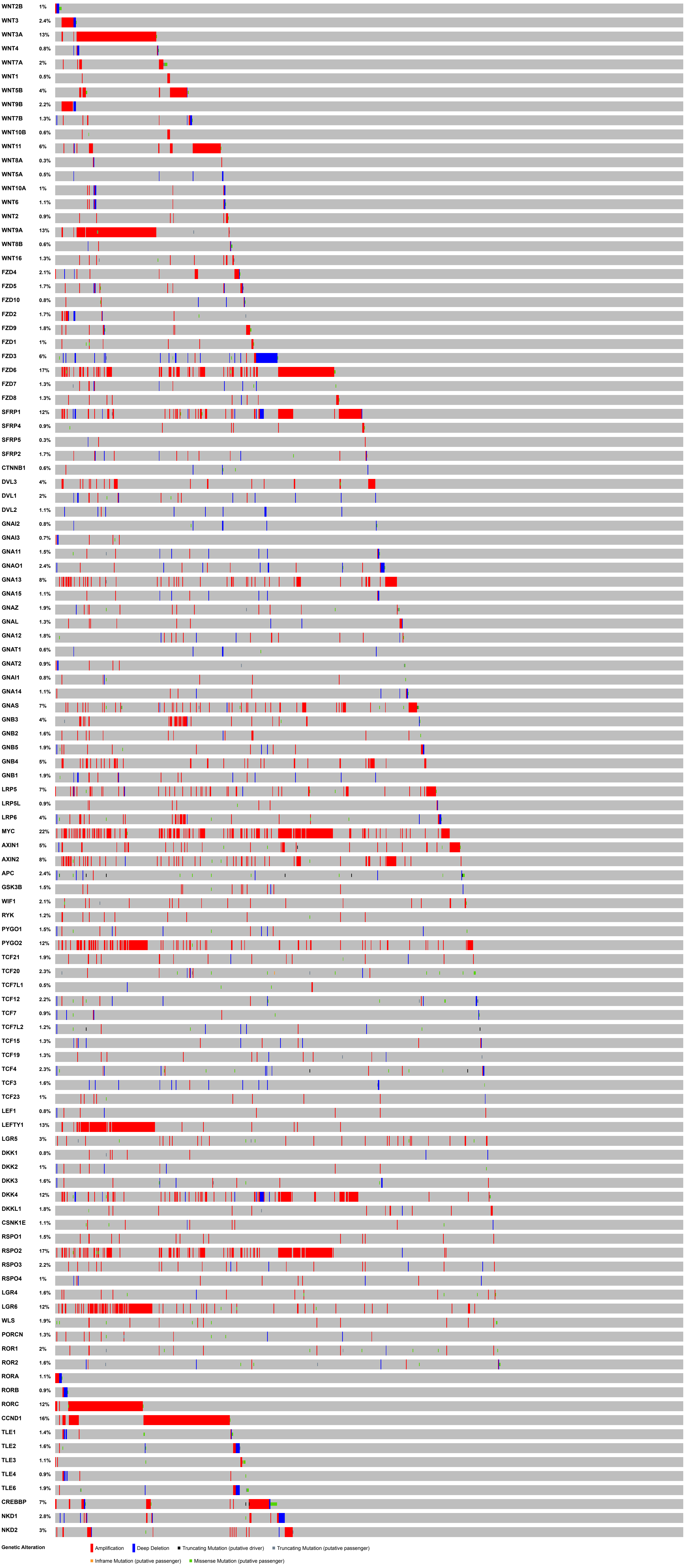

Cluster #4-dependent		Cluster #1-dependent		Non-clustered	
name	Degree	name	Degree	name	
FZD7	50	VIM	56	APOE	LILRB1
LEF1	46	TEK	55	ARHGAP4	MAP4K2
IRX3	46	FZD4	53	AXIN2	MET
KLF5	46	TCF4	45	BIRC5	MMP1
SOX9	45	WISP2	43	CCND1	MMP9
MMP7	40	COL8A1	42	CDC25B	MSL1
TCF7	40	PEPD	42	CDKN2A	MSLN
CDH1	40	ELN	41	CTLA4	MYC
BTRC	40	PROCR	40	CXCL1	MYCN
TBX3	39	ENPP2	36	CYP1A1	NANOG
TGIF1	39	TNFSF9	36	DKK1	NKX2-2
RUNX2	39	FGF18	34	DLL1	NOS2
STRA6	38	MITF	33	DUSP6	NRCAM
LBH	38	FN1	31	EDN1	PDCL
HES1	36	LMO2	30	EN2	PITX2
MYCBP	33	VEGFC	29	FGF20	PLCG2
RBP1	30	EFNB1	27	FGF9	RET
ABCB1	26	IGF1	26	FST	RHOU
CLDN1	21	MMP2	26	GJA1	SALL4
FOXN1	20	NOS3	25	GJB6	SIX3
CACNA1G	17	TWIST1	22	ID2	SOX2
RARG	14	VCAN	19	IFI30	SP5
TNFSF11	10	GPNMB	19	IL1B	TERT
VEGFA	9	IGF2	18	IL32	TIAM1
UBE2I	9	ISLR	18	ISL1	TNFRSF11B
CD44	8	EDA	16	ITGB7	TSHR
ENC1	7	SOX17	15	JUN	WNT3A
PTTG1	7	IL6	15	L1CAM	LGR5
POU5F1	7	CREM	13		
GREM2	6	SNAI1	13		
MMP3	6	BMP4	10		
CDX1	3	SFRP2	9		
SIAH2	2	PLAUR	9		
POSTN	2	CYBB	9		
BGLAP	1	CYR61	8		
PPARD	1	FOSL1	8		
		DUSP1	7		
		FOS	6		
		PTGS2	6		
		JAG1	5		
		KLF10	4		
		EGFR	3		
		TNFRSF4	2		
		WISP1	2		

Supplementary Table 1. Clusters of Wnt target genes showing strong correlations with the Wnt ligands and other components of the Wnt pathway.

Subgroup 1		Subgroup 2	
Name	Node degree	Name	Node degree
WNT7B	41	WNT7A	40
WNT5B	40	WNT5A	40
WNT5A	24	WNT2	29
WNT7A	24	WNT6	21
FZD6	5	FZD4	37
FZD2	40	FZD6	29
FZD4	26	FZD7	23
FZD1	22	GNA14	37
GNAI2	46	GNAL	30
GNA13	43	DVL1	30
DVL1	43	GNB2	29
GNB2	42	GNA13	29
NKD2	38	SFRP4	41
SFRP4	43	RORA	38
DKK3	36	DKK2	35
SFRP2	34	SFRP2	31
DKK2	32	DKK3	30
ROR2	41	RORB	31
LRP6	35	ROR1	30
RYK	34	RSPO1	24
GSK3B	42	APC	42
APC	41	TCF7	34
WLS	38	MYC	34
AXIN2	37	AXIN2	33
LEF1	33	PYGO2	32

Supplementary Table 2. Wnt pathway component genes, which show high degree of connectivity in the correlation networks for the TNBC subgroups.

Interactions for the whole TNBC cohort	
SFRP2 (interacts with) SFRP4	0.63
DVL1 (interacts with) GNB2	0.62
FZD4 (interacts with) GNA14	0.60
FZD7 (interacts with) LRP6	0.59
DKK3 (interacts with) SFRP2	0.58
FZD2 (interacts with) GNAI2	0.56
DKK3 (interacts with) SFRP4	0.52
FZD4 (interacts with) SFRP4	0.52
GNA13 (interacts with) GNB2	-0.52
FZD7 (interacts with) SFRP1	0.51
GNA13 (interacts with) RORA	0.51
GNA14 (interacts with) WNT9B	0.48
FZD4 (interacts with) WNT9B	0.48
GNA13 (interacts with) GNB4	0.47
GNB2 (interacts with) LRP6	-0.46
GNB5 (interacts with) WNT7A	0.46
DKK3 (interacts with) FZD4	0.45
GNA15 (interacts with) WNT7A	0.45

Interactions in the subgroup 1	
FZD4 (interacts with) WNT9B	0.80
ROR2 (interacts with) WNT5B	0.79
DVL1 (interacts with) GNB2	0.73
DKK2 (interacts with) GNAO1	0.72
FZD6 (interacts with) GNA13	0.71
GNA13 (interacts with) GNB2	-0.71
FZD2 (interacts with) GNAI2	0.70
DKK3 (interacts with) WNT5B	0.70
FZD7 (interacts with) LRP6	0.69
GNA14 (interacts with) WNT9B	0.68
GNAO1 (interacts with) NKD1	0.68
DKK3 (interacts with) SFRP4	0.67
GNB4 (interacts with) WNT5B	-0.67
GNAI2 (interacts with) ROR2	0.67
FZD7 (interacts with) SFRP1	0.67
FZD6 (interacts with) SFRP1	0.66
DKK3 (interacts with) SFRP2	0.66
GNA13 (interacts with) GNB4	0.65
GNAI3 (interacts with) WNT9B	-0.65
GNB2 (interacts with) LRP6	-0.65
DKK3 (interacts with) ROR2	0.65
DKK2 (interacts with) WNT5A	0.64
FZD4 (interacts with) GNA14	0.64
GNAI2 (interacts with) SFRP4	0.64
GNA12 (interacts with) GNAO1	0.64
RSPO1 (interacts with) SFRP2	0.64
GNAI2 (interacts with) NKD2	0.64
GNA12 (interacts with) GNAI2	0.64
SFRP2 (interacts with) SFRP4	0.63
GNA13 (interacts with) RYK	0.63
GNAI2 (interacts with) GNB4	-0.63
GNAI2 (interacts with) WNT5B	0.63
FZD7 (interacts with) GNB2	-0.63
GNAL (interacts with) RSPO1	0.62
GNAI3 (interacts with) RYK	0.61
ROR2 (interacts with) SFRP4	0.61
FZD2 (interacts with) GNA13	-0.61
GNA13 (interacts with) GNAI2	-0.61
GNB5 (interacts with) WNT7A	0.61
DKK4 (interacts with) ROR1	0.61
GNB2 (interacts with) SFRP1	-0.60
GNA13 (interacts with) SFRP4	-0.60
GNAI2 (interacts with) GNB2	0.60

Interactions in the subgroup 2	
SFRP4 (interacts with) WNT2	0.77
FZD4 (interacts with) GNA14	0.70
DKK2 (interacts with) RORA	0.70
FZD4 (interacts with) SFRP4	0.69
GNA14 (interacts with) SFRP4	0.67
DKK2 (interacts with) FZD4	0.66
GNA14 (interacts with) RORA	0.65
GNA13 (interacts with) RORA	0.65
FZD4 (interacts with) RORA	0.64
SFRP2 (interacts with) SFRP4	0.64
DKK2 (interacts with) DVL1	-0.64
RORA (interacts with) WNT2	0.64
GNB2 (interacts with) RORA	-0.64
GNA14 (interacts with) GNB5	0.62
DKK3 (interacts with) FZD4	0.62
DKK2 (interacts with) SFRP4	0.61
DKK2 (interacts with) GNA14	0.61
RORA (interacts with) WNT7A	0.61
SFRP2 (interacts with) WNT2	0.60
GNB2 (interacts with) WNT7A	-0.60
GNA14 (interacts with) WNT2	0.60

Supplementary Table 3. Most prominent pairwise interactions in TNBC and its subgroups. In green, the pairs with physical interactions (either known or inferred from orthologies) are highlighted in green. In blue, negative correlations, which are highly unlikely in the case of physical interactions, are highlighted.
